# Management of epilepsy in the brain tumour patient: Current concepts and practical approaches

**DOI:** 10.1016/j.clinme.2026.100650

**Published:** 2026-09-28

**Authors:** Jeremy Rees

**Affiliations:** a Department of Neurology, Shaarei Zedek Medical Center, Jerusalem, Israel; b National Hospital for Neurology and Neurosurgery, Queen Square and Cleveland Clinic London, United Kingdom; c Queen Square Institute of Neurology, University College London, United Kingdom

**Keywords:** Brain tumour, Epilepsy, Seizures, Anti-seizure medications, Surgery, Radiotherapy, Chemotherapy, Vagal nerve stimulator, Laser interstitial thermal therapy

## Abstract

Brain tumour-related epilepsy (BTRE) is among the most common, disabling and feared complications of primary and metastatic brain tumours. Seizures cause significant suffering, psychosocial burden, reduced quality of life and lead inevitably to the loss of a driving license. The mechanisms underlying BTRE are multifactorial, including tumour-driven alterations in neuronal excitability and pro-epileptogenic metabolites. Management is more challenging than idiopathic epilepsy due to high rates of drug resistance, the natural history of brain tumours and potential interactions between anti-seizure medications (ASMs) and chemotherapy/steroids. This review summarises the pathophysiology of BTRE, outlines risk factors, and provides practical recommendations for ASM selection, prophylaxis, withdrawal, and the role of surgery, radiotherapy, chemotherapy, and neuromodulation. Updated clinical evidence supports the use of non-enzyme-inducing ASMs as first line treatment as these do not interact with chemotherapy/steroids and are better tolerated than the first generation ASMs. ASM prophylaxis is not recommended and ASM withdrawal, particularly in patients with gliomas, should be made only after careful consideration of the patient's seizure history and the stability of their disease.

## Introduction

Brain tumour–related epilepsy (BTRE) is defined as one or more unprovoked seizures due to the presence of a brain tumour and has profound consequences for independence, driving, employment, and psychosocial wellbeing.

As epilepsy is a cortical disease, supratentorial tumours cause seizures – many gliomas occur in the frontal, temporal and insular lobes which are particularly epileptogenic areas of the brain. Seizures are extremely uncommon in people with posterior fossa or deep-hemispheric tumours. A recent retrospective review reported two out of 44 patients with cerebellar meningiomas presenting with seizures.^1^ Often BTRE is the first presenting symptom of a brain tumour but can occur at any point throughout the disease course.

There is an inverse relationship between the tumour grade and the likelihood of BTRE. Seizures occur in up to 85% of patients with low-grade gliomas and 50–70% of those with high-grade gliomas.^2^ In contrast, only about 30% of patients with meningiomas^3^ and 10–12% of patients with Brain Metastases develop seizures.^4^ Up to 70% of patients experience seizures at the end of life.^5^

The management of BTRE is uniquely complex. Tumour biology, peritumoural microenvironment and clinical factors all influence seizure risk and response to therapy. Moreover, ASMs may interact with chemotherapeutic agents and corticosteroids, restricting the range of potential drug options.

This review provides a contemporary overview of BTRE with a focus on the practical clinical considerations and management.

## Pathophysiology of BTRE

The epileptogenicity of brain tumours arises from a combination of structural, metabolic, and inflammatory mechanisms:

### Peritumoural glutamate excess

Glutamate plays an important role in the pathophysiology of BTRE by accumulating in the extracellular space, where it drives both tumour progression and the hyper-excitability of surrounding brain tissue that triggers seizures.^6^ GBM cells take in cystine and, in turn, release abnormally high concentrations of glutamate into the surrounding brain microenvironment via the cystine–glutamate antiporter (system X_c_).^7^

### IDH mutations and D-2-hydroxyglutarate (D-2HG)

Isocitrate dehydrogenase (IDH) is a critical enzyme in the Krebs cycle. IDH1 and IDH2 mutations are found in both low-grade and high-grade gliomas and give the enzymes a ʻgain-of-function’ that forces them to convert alpha-ketoglutarate into the oncometabolite D-2-hydroxyglutarate (D-2HG) rather than isocitrate. The accumulation of D-2HG, which is structurally similar to glutamate, acts as an excitatory agonist at NMDA receptors. This may help to explain the high seizure rates in IDH-mutant low-grade gliomas.^8^, ^9^ IDH mutations serve as a critical diagnostic and prognostic biomarker in diffuse gliomas, defining a distinct molecular subtype that typically occurs in younger patients and is associated with a significantly better survival compared to IDH-wildtype tumours.^10^

### Peritumoural inflammation and haemorrhage

Microglial activation, cytokine release (IL-1β, TNF-α), and intratumoural haemorrhage all lower seizure threshold.^11^

## Risk factors for BTRE

Risk factors for BTRE include the anatomical location of the tumour (particularly the cortical and temporal/frontal regions), tumour grade and histology (particularly glioneuronal tumours and low-grade diffuse gliomas), and the presence of the IDH1/2 mutations (see above). Table 1 outlines the most common associations with BTRE.

**Table 1:** Risk factors for BTRE.

Category	Key risk factors
**Primary brain tumours**	Low grade gliomas; oligodendroglial histology; cortical location; frontal/temporal involvement; IDH mutation
**Brain metastases**	multiple metastases; supratentorial location
**Treatment related**	Radiation treatment; Immediate postoperative period
**Patient related**	Younger age; longer survival

## General principles

Neurologists who manage BTRE need to be aware of the following:

• Ensure the diagnosis of epilepsy is correct and rule out mimics such as migraine and non-epileptic attack disorder
• Be familiar with the newer ASMs, aiming to minimise adverse effects and drug-drug interactions with chemotherapy and corticosteroids
• Ensure the patient understands the implications of BTRE, particularly the restrictions on driving
• Provide a reliable contact point for answering questions and concerns outside of the outpatient setting

Management requires close collaboration between the neurologist and other colleagues within the neuro-oncology MDT.

## Anti-seizure medications (ASMs)

### Starting ASMs

All patients with BTRE should be started on ASMs after the **first** seizure given the high recurrence risk (in contrast to the general population where ASMs are usually started after the second seizure). Both focal and generalised seizures warrant treatment.^12^

Unlike the older ASMs, none of the newer ASMs induce the CYP450 enzyme system and therefore do not interfere with corticosteroid dosing or with chemotherapy agents, such as temozolomide or lomustine, used for primary brain tumours or with immunotherapy used for certain Brain Metastases.

The most common first line-ASMs are levetiracetam, lamotrigine. Both are non-enzyme inducing ASMs and licensed as monotherapy for the treatment of focal-onset seizures with and without secondary generalisation.

Levetiracetam (LEV) has the advantage of rapid titration (a therapeutic dose of 500 mg bd can be started immediately) with high efficacy (up to 90% seizure freedom in BTRE) and a good side-effect profile.^13^ It should be avoided in people with a previous history of anxiety and depression as it can cause mood disorders, anxiety and irritability (Keppra rage). Patients who achieve optimal seizure control with levetiracetam but suffer from intolerable neuropsychiatric or behavioural side effects, such as severe irritability, aggression, or mood disturbances, can be switched overnight to brivaracetam but at a tenth of the dose.^14^

Lamotrigine (LTG) has to be titrated more slowly but has a good long-term retention profile. Because of the need for slow titration, it is not the preferred first line drug when immediate risk reduction of seizure recurrence is required. The most important side-effect to warn the patient about is a rash which can progress to Stevens-Johnson syndrome and mandates immediate cessation. It does not cause mood disturbance.

Lacosamide (LAC) is also effective for BTRE^15^ but is only licensed in the UK as add-on therapy for focal onset seizures (in contrast to the USA and Canada where it is licensed as monotherapy.)

There are no direct comparisons between levetiracetam, lamotrigine and lacosamide for BTRE but a recent retrospective study found an overall 12-month seizure freedom of 64.9% and retention rate of 79.7%. There were no significant differences in seizure freedom between the three drugs, reflecting our clinical experience.^16^

For a more detailed discussion of the pathophysiology and treatment of BTRE, the reader is recommended a recent consensus review from the Society of Neuro-oncology.^17^

### When to add a second drug

When monotherapy fails, either because the first drug was ineffective despite being at a therapeutic dose or because it caused intolerable side-effects, the patient should be switched to a second monotherapy. Ideally only one medication should be used initially, to minimise the risk of drug interactions and side effects. However, there may be a case for adding a second ASM before achieving a maximum dose – in a retrospective study of patients on LEV as first-line therapy, adding another ASM was more effective than simply increasing the LEV dose^18^ (see Fig. 1).

**Fig. 1:**
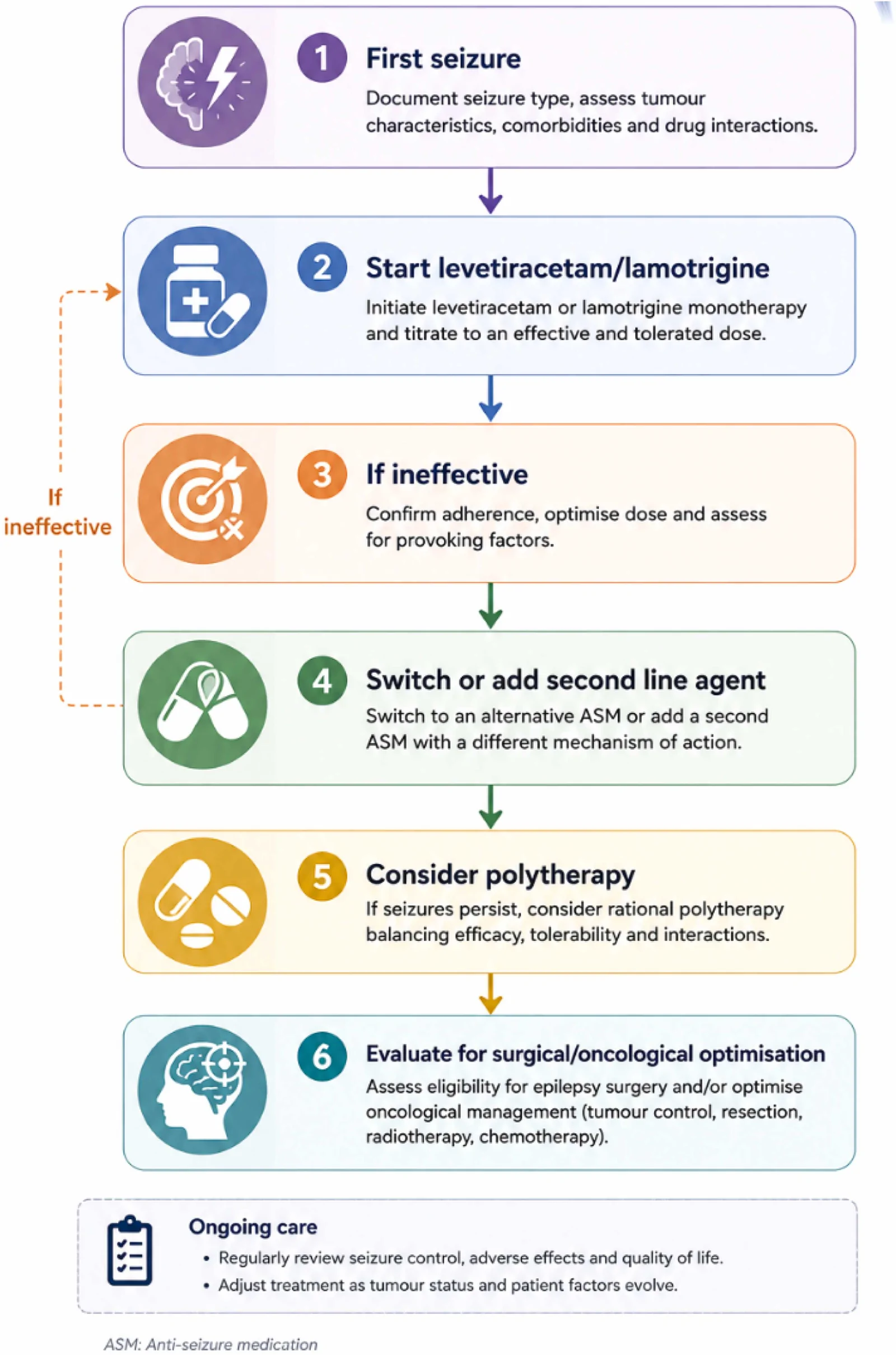
Algorithm for seizure management in BTRE.

### Second line ASMs

There are numerous second-line ASMs for BTRE but the evidence for superiority of one drug over another is not available. The following are the more frequently used drugs in current use and some salient points when prescribing:

• **Lacosamide** – It can prolong the PR interval, which can rarely lead to heart block or bradycardia, so a baseline ECG should be done particularly in older patients.
• **Perampanel** – Patients may develop neuropsychiatric symptoms (such as aggression, irritability, and anger), severe dizziness, or extreme sleepiness.
• **Cenobomate** – A relatively newer drug which I have found to be useful in patients with intractable BTRE. Requires slow titration to avoid Drug Related Eosinophilia with Systemic Symptoms (DRESS).
• **Topiramate** is an enzyme-inducing agent and should be avoided in women of childbearing potential. Annual Risk Assessment Forms are required in the UK.
• **Sodium Valproate (valproic acid)** is an effective ASM and may have a role in directly inhibiting tumour growth through its action as a Histone Deacetylase (HDAC) Inhibitor, modifying the epigenetic structure of the tumour cells. It does not interact with chemotherapy. However, it has become very difficult to prescribe due to its teratogenicity in women and a potential small increased risk of neurodevelopmental disorders (such as autism) in children conceived by fathers taking the medication, as well as historical concerns regarding potential effects on male fertility. Annual Risk Assessment Forms are required in the UK.
• **Clobazam** – Particularly useful when patients are experiencing a flare-up of seizures. It is less commonly used for chronic epilepsy as there is a high rate of tolerance.

Levetiracetam and sodium valproate are available both intravenously and as a liquid formulation which makes them useful in paediatric patients and those who have lost the ability to swallow towards the end of life.

## ASM prophylaxis

As randomised trials and meta-analyses show no reduction in early or late seizures with prophylactic ASMs, routine prophylaxis in seizure-naïve patients is **not recommended**.^19^ Some neurosurgeons use levetiracetam peri-operatively, but evidence for long-term benefit is lacking.

## ASM withdrawal

It is generally recommended that ASM withdrawal in BTRE is only considered after achieving at least two years of complete seizure freedom following gross total resection of a low-grade gliomas and only when the imaging is stable.

This is because the risk of seizure recurrence in glioma patients after ASM withdrawal is high. In a prospective multicenter study, the cumulative incidence of a recurrent seizure at 48 and 96 months was 48% and 66% for the ASM withdrawal group vs 28% and 52% for the ASM continuation group.^20^ Therefore, patients who wish to come off their ASM need to be made fully aware of the increased risks and in particular the driving guidelines. In the UK, a patient who has a recurrent seizure after a period of seizure freedom, even a focal non-motor aware seizure, will have to surrender their license for at least a year.

## Tumour treatments and seizure control

All oncological treatments improve seizure control, irrespective of the type of seizure.

### Surgery

Surgical resection is the most effective intervention for BTRE.^21^ Gross-total resection improves seizure freedom rates in up to 90% of patients with low-grade gliomas.^22^ Early surgery is associated with better long-term seizure control.

### Radiotherapy and chemotherapy

Both radiotherapy and chemotherapy reduce seizure frequency in low-grade gliomas.^23^ A retrospective study has shown that 76% of patients achieve ’significant’ (≥50%) seizure reduction at one year after radiotherapy. Timing of radiotherapy and duration of seizures prior to radiotherapy were significantly associated with seizure reduction.^24^ Rarely patients can have an exacerbation of seizures during or shortly after the course of radiation.

There are very few prospective studies examining the effect of chemotherapy alone on BTRE but there are no reports of chemotherapy exacerbating seizure control. A recent single institution study of 39 patients with Grade 2 oligodendrogliomas treated with temozolomide showed a reduction in seizure frequency in 90% of patients, independent of ASM regimen adjustments or prior antitumor treatment. In 41% the ASM dose was successfully reduced and 26% were able to stop ASMs completely.^25^

## Non-pharmacological approaches

### Vagus nerve stimulation (VNS)

Vagus nerve stimulation (VNS) is an established adjunctive therapy for patients with medically intractable epilepsy. Targeted electrical impulses are delivered via a stimulator to the left vagus nerve causing desynchronisation of abnormal electrical cortical activity and an increase in seizure threshold. VNS is particularly beneficial for patients with BTRE whose tumour is stable and who have already had tumour surgery or are not a candidate for further surgery.^26^ Even though it rarely eliminates seizures, it does provide significant long-term seizure reduction and can also decrease the severity and duration of breakthrough seizures. Patients who experience a warning prior to their seizures can swipe the stimulator using a magnet, thus delivering a burst of electrical stimulation on demand and offering proactive protection against seizure development. The main practical issue is that MRI compatibility requires specialist centres who can turn off the stimulator prior to the scan.

### Laser interstitial thermal therapy (LITT)

There is emerging evidence from small retrospective case series that suggest a beneficial effect of LITT in selected patients with deep-seated or recurrent gliomas.^27^

## Special considerations

### Pregnancy

Both levetiracetam and lamotrigine are safe in pregnancy and breastfeeding. In contrast topiramate and sodium valproate should be avoided. The risk of pregnancy on tumour growth is low except for meningiomas. In a literature review of 148 cases of pregnancy-associated meningiomas, four risk factors were identified – parasellar location, anterior circulation blood supply, visual symptoms at presentation and a high rate of clear-cell and chordoid morphology.^28^

### Cognitive effects

Most of the newer ASMs have a good cognitive profile. It is advisable to avoid topiramate and high-dose polytherapy particularly if the patient already has cognitive impairment as a result of the tumour or the previous radiotherapy.

### Hepatic and renal insufficiency and geriatric populations

In older patients with BTRE and those who have renal and/or hepatic impairment, the standard first-line ASMs are generally regarded as safe in these populations although dosage reduction is sometimes required for levetiracetam and lacosamide in patients with severe renal impairment.

## Driving considerations in the UK

Driving restrictions in patients with brain tumours are highly individualised and guided both by the risk of seizures, the tumour grade and histology, the binocular visual acuity, the presence of visual field defects, psychomotor slowing and any residual physical or cognitive impairments. Most jurisdictions require patients to stop driving for at least 6–12 months following a craniotomy or the onset of seizure activity to ensure clinical stability. In the UK, the DVLA (Driver and Vehicle Licensing Agency) have drawn up comprehensive and updated guidelines for all types of brain tumour in the **Assessing Fitness to Drive.**^29^

## Conclusion

BTRE remains a major source of morbidity in neuro-oncology. Both levetiracetam and lamotrigine are effective first-line ASMs, but up to half of patients require combination therapy. Surgical resection, radiotherapy, and chemotherapy all improve seizure control. Despite progress, high-quality prospective trials are urgently needed to refine treatment strategies and improve outcomes for patients with BTRE.

## Key points

• **Levetiracetam or lamotrigine** should be offered as first-line therapy for BTRE due to efficacy and minimal interactions and are safe in pregnancy and breastfeeding.
• **Prophylactic ASMs are not recommended** for seizure-naïve patients with brain tumours.
• **ASM withdrawal carries a high relapse risk** and should be considered only in patients who have had Gross Total Resections, stable disease and at least two years seizure freedom.
• **Surgery, radiotherapy, and chemotherapy** all improve seizure control.
• **Vagus nerve stimulation** is a useful adjunct in selected patients with intractable BTRE.
• **Driving restrictions** are guided not just by the BTRE but also by the location, subtype and grade of the tumour and the neurological impairments caused by the tumour and its treatment.

## CRediT authorship contribution statement

**Jeremy Rees:** Conceptualization, Writing – original draft, Writing – review & editing.

## Declaration of generative AI and AI-assisted technologies in the writing process

During the preparation of this work, the author used Chat GPT for designing and producing Fig. 1. He has reviewed and edited the output as needed and takes full responsibility for the content of the published article.

## Funding

The author has not received any funding towards the preparation of this manuscript.

## Declaration of competing interest

The author declares that he has no known competing financial interests or personal relationships that could have appeared to influence the work reported in this paper.

## References

[1] Goertz L., Bernards N., Muders H. (2024). Incidence and clinical presentation of pre- and postoperative seizures in patients with posterior fossa meningiomas. Cureus.

[2] Lote K., Stenwig A.E., Skullerud K., Hirschberg H. (1998). Prevalence and prognostic significance of epilepsy in patients with gliomas. Eur J Cancer.

[3] Englot D.J., Magill S.T., Han S.J. (2016). Seizures in supratentorial meningioma: a systematic review and meta-analysis. J Neurosurg.

[4] Lamba N., Catalano P.J., Cagney D.N. (2021). Seizures among patients with brain metastases: a population- and institutional-level analysis. Neurology.

[5] Brauchitsch S.V., Strzelczyk A., Rosenow F. (2022). High end-of-life incidence of seizures and status epilepticus in patients with primary and secondary brain tumors. J Neurooncol.

[6] Wong M. (2012). Tumors generate excitement: the role of glutamate in tumor-related epilepsy. Epilepsy Curr.

[7] Venkataramani V., Tanev D.I., Strahle C. (2019). Glutamatergic synaptic input to glioma cells drives brain tumour progression. Nature.

[8] Mortazavi A., Fayed I., Bachani M (2022). IDH-mutated gliomas promote epileptogenesis through d-2-hydroxyglutarate-dependent mTOR hyperactivation. Neuro Oncol.

[9] Divé I., Luger A.-L., Muench D. (2025). State of the art: glioma-associated epilepsy - bridging tumor biology and epileptogenesis. Neurol Res Pract.

[10] The Cancer Genome Atlas Research Network (2015). Comprehensive, integrative genomic analysis of diffuse lower-grade gliomas. N Engl J Med.

[11] Vezzani A., Balosso S., Ravizza T. (2019). Neuroinflammatory pathways as treatment targets and biomarkers in epilepsy. Nat Rev Neurol.

[12] van Breemen M.S., Wilms E.B., Vecht C.J. (2007). Epilepsy in patients with brain tumours: epidemiology, mechanisms, and management. Lancet Neurol.

[13] Zhang Y., Li H., Zhang B., Zhang J., Li C. (2025). Efficacy and safety of levetiracetam in preventing postoperative seizures in adult patients with brain tumors: a meta-analysis. Front Neurol.

[14] Abraira L., Salas-Puig J., Quintana M. (2021). Overnight switch from levetiracetam to brivaracetam. Safety and tolerability. Epilepsy Behav Rep.

[15] Rudà R., Houillier C., Maschio M. (2020). Effectiveness and tolerability of lacosamide as add-on therapy in patients with brain tumor–related epilepsy: results from a prospective, noninterventional study in European clinical practice (VIBES). Epilepsia.

[16] Tabrizi N., Frouzanian M., Shafiee S., Alizadeh-Navaei R. (2026). Real-world evidence on the use of new antiseizure medications in brain tumor-related epilepsy. Epilepsy Res.

[17] Avila E.K., Tobochnik S., Inati S.K. (2024). Brain tumor-related epilepsy management: a Society for Neuro-oncology (SNO) consensus review on current management. Neuro Oncol.

[18] Hattori E.Y., Arakawa Y., Mineharu Y. (2023). Seizure control by adding on other anti-seizure medication on seizure during levetiracetam administration in patients with glioma-related epilepsy. BMC Cancer.

[19] Walbert T., Harrison R.A., Schiff D. (2021). SNO/EANO guideline update on anticonvulsant prophylaxis. Neuro Oncol.

[20] van der Meer P.B., Kerkhof M., Dirven L. (2025). Long-term follow-up results of antiseizure medication withdrawal in grade 2 and 3 glioma patients: a prospective observational study. Neurology.

[21] Khalili B.F., Chojnacki M.R., Dixit K. (2025). Surgical treatment of brain tumor-related epilepsy: current and emerging strategies. Cancers.

[22] Chang E.F., Potts M.B., Keles G.E. (2008). Seizure characteristics and control following resection in 332 patients with low-grade gliomas. J Neurosurg.

[23] Koekkoek J.A., Kerkhof M., Dirven L. (2015). Seizure outcome after radiotherapy and chemotherapy in low-grade glioma patients: a systematic review. Neuro Oncol.

[24] Rudà R., Magliola U., Bertero L. (2013). Seizure control following radiotherapy in patients with diffuse gliomas: a retrospective study. Neuro Oncol.

[25] Haggiagi A., Avila E.K. (2019). Seizure response to temozolomide chemotherapy in patients with WHO grade II oligodendroglioma: a single-institution descriptive study. Neurooncol Pract.

[26] Patel K.S., Moussazadeh N., Doyle W.K. (2013). Efficacy of vagus nerve stimulation in brain tumor-associated intractable epilepsy and the importance of tumor stability. J Neurosurg.

[27] Nielsen S.H., Rasmussen R. (2024). MR-guided laser interstitial thermal therapy in the treatment of brain tumors and epilepsy. Acta Neurochir.

[28] Laviv Y., Ohla V., Kasper E.M. (2018). Unique features of pregnancy-related meningiomas: lessons learned from 148 reported cases and theoretical implications of a prolactin modulated pathogenesis. Neurosurg Rev.

[29] Driving Vehicle Licensing Authority. Assessing fitness to drive: guide for medical professionals. Neurological Disorders.

